# Hallucinogen Persisting Perception Disorder in a Young Adult Case Report

**DOI:** 10.1155/crps/6605265

**Published:** 2026-09-03

**Authors:** Sonali Notani, Vikas Srinivasa, Serena Chaudhry, Ashley Weiss

**Affiliations:** ^1^ Tulane University School of Medicine, New Orleans, Louisiana, USA, tulane.edu

**Keywords:** bipolar, case report, hallucinogen persisting perception disorder (HPPD), LSD, psychosis

## Abstract

Hallucinogen persisting perception disorder (HPPD) is a rare psychiatric diagnosis encompassing a range of perceptual disturbances caused by the lingering effects of hallucinogens. There are two major subtypes of the disorder: HPPD I and II. HPPD II is considered a more severe form that is generally longer‐term, distressing, and requiring more intense treatment. In the limited available literature, many of the affected patients have multiple psychiatric comorbidities. The following case describes a 24‐year‐old college student, WA, who was diagnosed with HPPD II along with comorbid bipolar I disorder and generalized anxiety disorder. WA presented with an escalation of manic symptoms, psychosis, and two lysergic acid diethylamide (LSD) uses prior to hospitalization. He was treated with olanzapine, leading to mood stabilization and remission of psychosis. Post treatment, WA continued to experience perceptual disturbances and “visual snow.” Medications clonidine and clonazepam were trialed for HPPD. While symptoms fluctuated in severity, they never fully resolved. Despite this, WA achieved acceptance of his symptoms, mood stability, and a functional recovery. WA’s case is an example of the clinical complexity in accurate diagnosis and treatment of HPPD, including the distinction of the symptoms from psychosis. As LSD‐assisted therapies continue to be investigated for conditions such as anxiety and depression, recognition and management of persistent adverse effects such as HPPD are becoming increasingly important in both psychiatric and general medical practice. This case demonstrates the limited effectiveness and lack of knowledge on HPPD treatments as well as the need for longitudinal assessments in management.

## 1. Introduction

Hallucinogen persisting perception disorder (HPPD) is a rare psychiatric diagnosis that is poorly understood due to limited cases. The condition is often diagnosed in individuals with other psychiatric comorbidities or substance misuse [1]. The effects of hallucinogens, referred to as “psychedelics,” include alterations in perception, consciousness, cognitive and sensory processing, and emotional volatility. Some users have reported a reduced sense of self‐awareness or “ego‐dissolution” [2]. Healers and shamans have been known to use these substances for thousands of years for religious and ritualistic purposes. The western culture of psychedelic use began in the late 1930s when lysergic acid diethylamide (LSD) was synthesized, one of the more commonly taken hallucinogenic substances. Currently, hallucinogens are taken socially or individually for their perception‐changing effects and self‐experience [1]. The use of these substances has been linked to major health and psychiatric complications.

According to the DSM‐5, HPPD is characterized by the recurrence of one or more perceptual symptoms that were previously experienced during hallucinogen intoxication following cessation of substance use. Common manifestations include geometric hallucinations, false perceptions of movement in the peripheral visual fields, flashes of color, intensified colors, trailing phenomena, positive afterimages, halos around objects, macropsia, and micropsia. These symptoms must cause clinically significant distress or impairment and cannot be attributable to another medical condition, psychiatric disorder, or substance‐related condition [3].

There are two major subtypes of hallucinogenic recurring perceptual disturbances, which have been categorized as HPPD I and II. HPPD I, or the benign flashback type, is considered a shorter‐term, nondistressing reversible state with a pleasant effect. Whereas HPPD II is a longer‐term and distressing, slowly reversible, or irreversible state with an unpleasant effect. Type II has a worse prognosis, with some patients requiring frequent treatment [4, 5]. Commonly prescribed medications are alpha‐2 agonists, such as clonidine, SSRIs, benzodiazepines, and mood stabilizers [1]. Within these two categories, there can be variation in the symptoms, onset, visual, frequency, duration, and severity of the perceptual disturbances. This disorder can also be associated with the onset of “auras” or feelings of detachment or derealization. The most reported symptoms are visual disturbances and hallucinations [4].

LSD is the most frequently attributable drug for HPPD; however, others include psilocybin, mescaline, cannabis, 5‐MeO‐DiPT, MDMA, PCP, dextromethorphan, and ketamine [4]. The neurobiological mechanisms underlying HPPD remain incompletely understood. One prevailing hypothesis suggests that hallucinogen exposure may lead to chronic disinhibition of visual processing pathways through the dysfunction of cortical serotonergic and GABAergic inhibitory systems. Specifically, LSD‐mediated disruption of inhibitory interneurons may impair the brain’s ability to appropriately filter sensory stimuli, resulting in persistent visual phenomena such as visual snow, halos, afterimages, and trailing effects. Other proposed mechanisms involve abnormalities within the lateral geniculate nucleus and related thalamocortical circuits responsible for visual processing and sensory gating. Recent reviews have also emphasized the heterogeneity of HPPD presentation, suggesting that multiple neurobiological mechanisms may contribute to symptom development and persistence. The nosological classification of HPPD also remains an area of ongoing debate. Clinical presentations range from transient, benign perceptual disturbances to chronic and functionally impairing syndromes, contributing to challenges in diagnosis, prognosis, and treatment selection [1, 6].

There have also been several comorbidities reported in patients with HPPD, including major depressive disorder, anxiety, bipolar disorder, dissociation, and schizophrenia spectrum disorders. In particular, HPPD II has been linked to a negative mood and affect [1, 6].

Interest in therapeutic applications of LSD has re‐emerged in recent years, with multiple clinical trials investigating LSD‐assisted therapy for generalized anxiety disorder, depression, and anxiety associated with serious medical illness. Early studies have demonstrated potential clinical benefits, contributing to the growing interest in the integration of psychedelic‐assisted therapies into psychiatric practice. As the use of LSD expands in both research and community settings, understanding uncommon but potentially persistent adverse effects such as HPPD becomes essential [7, 8].

The presented case is one of the first in the literature describing HPPD II emerging during the treatment course of a first episode of psychosis associated with bipolar I disorder and underlying generalized anxiety disorder.

## 2. Case Presentation

WA is currently a 26‐year‐old male with a longstanding history of generalized anxiety disorder, bipolar I disorder, and HPPD II. In 2021, at the age of 20, WA experienced changes in mood and a rapid escalation of manic symptoms over 6 days with feelings of euphoria, increased strength, ambitiousness, and motivation, racing thoughts, psychosis, and a decreased need for sleep. His psychotic symptoms included a disorganized thought process and unusual beliefs about his body posture. This resulted in psychiatric hospitalization. WA had used LSD twice in the 6 weeks prior to being hospitalized and increasingly used marijuana over the prior year. While inpatient, he was found to have nontraumatic rhabdomyolysis, elevated CK, and hypokalemia associated with potential synthetic marijuana usage. His mental status while inpatient was significant for speech and thought disorganization with some bizarre beliefs about his physical body being distorted and visual hallucinations of “seeing things in lights.” He was given olanzapine 10 mg daily at the hospital, which led to remission of psychosis and mood stabilization. His psychosis was attributed to substance intoxication. After staying home with family for a couple months, he returned to college and began treatment at the local first‐episode psychosis (FEP) program for outpatient care.

Per history, WA met all developmental milestones and was academically gifted as a student. He reported always being sensitive emotionally and tended to worry more than his peers. WA always felt anxious about transitions and had a long‐standing sensitivity to sounds. In the context of family history of psychiatric illness, WA’s mother had a brief psychotic episode when she was 19, resulting in hospitalization but never developed a chronic psychotic disorder. WA was physically healthy and had no substance use prior to smoking marijuana a year before his psychotic episode, which he claims was precipitated by increasing social and generalized anxiety. WA also described seeing visual halos around lights and scattered visual disturbances that he could not pinpoint a cause for. After gathering a longitudinal history, the psychiatrist at the FEP program confirmed a diagnosis of bipolar I disorder and generalized anxiety disorder.

After his episode, WA was taking olanzapine; however, he complained of weight gain, low mood, intermittent suicidal ideation, and continued anxiety. He was abstaining from any marijuana or LSD use at the time as well. Olanzapine was cross‐titrated to lurasidone for a preferred side effect profile; this was eventually discontinued. More traditional mood stabilizers were also tried since the working diagnosis was a primary mood disorder with psychotic features. Lamotrigine was started, which led to a rash. He was then put on valproic acid, and it was titrated to therapeutic level. He was also prescribed clonazepam 1 mg in the evenings for temporary help with anxiety interfering with sleep onset. With this regimen, experienced stabilization in mood, reduced anxiety, and improved functioning in all aspects of life. However, throughout the course of all medication trials, WA continued to experience visual perceptual disturbances, which he called “visual snow.” He described seeing flashing lights that looked like “TV static.” One of the diagnostic challenges was differentiating these symptoms from bipolar disorder with psychotic features; however, he was having these disturbances even in the context of antipsychotic treatment. There were also no associated auditory hallucinations or other symptoms of psychosis. WA described seeing trailing lights as well as halos around lights. He reported no major interference with daily function other than having difficulty reading and feeling slightly distressed. He also denied any dissociation or detachment symptoms. His treatment plan evolved to include a diagnosis of HPPD, related to LSD use. The diagnostic assessment was based on longitudinal psychiatric history, serial mental status examinations, substance‐use history, and the persistence of characteristic visual disturbances after cessation of LSD and marijuana. His symptoms vacillated in intensity, some days disturbing him more than others. The visual snow was noted to decrease briefly in the evenings, corresponding to when he took clonazepam.

After graduation from college, WA’s treatment plan evolved to include specific treatment of HPPD symptoms. The decision was made to wean clonazepam and try clonidine based on the limited literature of it relieving HPPD symptoms. WA’s HPPD symptoms initially worsened after weaning clonazepam (his dose was reduced to 0.5 mg and then discontinued) but eventually reduced again with the addition of clonidine. His clonidine dose was increased over time to 0.1 mg three times a day, with an initial reduction in the intensity of visual snow; however, WA soon wanted to discontinue it due to sedation. Most recently, WA has begun taking escitalopram for anxiety with significant relief. He also continues to take valproic acid for mood stabilization. The patient’s clinical course and treatment history are summarized in Table 1. Currently, it has been 4 years since WA’s HPPD symptoms first appeared and his first experience with LSD. He recently shared in early 2025 that he had reached a level of acceptance with his HPPD symptoms and felt that he was better able to cope and function with them than he had in the past. He has been successful in graduating from the firefighting academy and has a job as a firefighter.

**Table 1:** Timeline of clinical presentation, treatment, and outcomes.

Time	Clinical events and findings	Intervention and outcome
2020–2021	Began using marijuana, with increasing use during the year preceding hospitalization.	—
Six weeks before hospitalization in 2021	Used LSD twice. Subsequently developed persistent visual disturbances, including visual snow, flashing or trailing lights, and halos around lights.	—
2021, age 20	Developed rapidly escalating manic symptoms over 6 days, including euphoria, racing thoughts, increased motivation, decreased need for sleep, disorganized thinking, unusual beliefs about his body, and visual hallucinations. He was hospitalized with nontraumatic rhabdomyolysis, elevated creatine kinase, and hypokalemia associated with possible synthetic marijuana use.	Treated with olanzapine 10 mg daily, resulting in remission of psychosis and mood stabilization. His psychosis was initially attributed to substance intoxication.
Several months later	Returned to college and established outpatient care through a first‐episode psychosis program. Longitudinal evaluation supported diagnoses of bipolar I disorder and generalized anxiety disorder. Visual disturbances persisted without other psychotic symptoms.	Continued olanzapine and remained abstinent from LSD and marijuana.
Subsequent outpatient treatment	Experienced weight gain, low mood, intermittent suicidal ideation, and continued anxiety while taking olanzapine.	Olanzapine was cross‐titrated to lurasidone, which was later discontinued. Lamotrigine was stopped after causing a rash.
Later outpatient course	Mood and anxiety improved, but visual snow, flashing lights, trailing lights, and halos continued despite antipsychotic treatment and in the absence of other psychotic symptoms.	Valproic acid was titrated to a therapeutic level. Clonazepam 1 mg nightly was added for anxiety and sleep, with a brief reduction in visual symptoms after each dose. HPPD II was diagnosed.
2022–2024	Treatment began to focus specifically on the persistent HPPD symptoms. Symptoms initially worsened during the clonazepam taper.	Clonazepam was reduced to 0.5 mg and then discontinued. Clonidine was initiated and increased to 0.1 mg three times daily, producing an initial reduction in visual snow. It was later discontinued because of sedation.
Early 2025	Continued to experience visual symptoms but reported greater acceptance and an improved ability to cope and function. He completed the firefighting academy and began working as a firefighter.	Continued valproic acid for mood stabilization. Escitalopram provided significant relief of anxiety.

## 3. Discussion

This case report is an example of the clinical complexity and diagnostic challenges associated with HPPD that occurred simultaneously with a first episode of psychosis associated with emerging bipolar disorder. WA’s presentation is unique and significant because of the persistence associated with his visual symptoms in the setting of resolving psychosis and mood stabilization and through trials of many medications that have some evidence in HPPD treatment.

Based on WA’s pharmacological outcomes and other available studies, there appears to be limited and inconsistent effectiveness of current treatment options for HPPD. While clonazepam seemed to provide symptom relief and help with anxiety, there are potential risks of dependence with long‐term use of it. Although clonidine is suggested as an effective treatment in prior reports, it proved to be ineffective for WA, with higher doses leading to side effects, making it intolerable. These observations showcase the need for more targeted research into HPPD pharmacotherapy as well as its interaction with mood stabilizers and antipsychotics in patients with co‐occurring psychiatric diagnoses.

Another limitation of this report is the lack of a standardized psychometric assessment throughout follow‐up. Changes in WA’s visual symptoms were documented through longitudinal clinical evaluation and patient self‐report rather than validated HPPD‐specific outcome measures. Consequently, the treatment response could not be quantified objectively, and subtle changes in symptom severity may have been missed. The development and routine use of validated instruments such as the HPPD Rating Scale may improve the precision of the outcome measurement.

Deciphering what symptoms were associated with which diagnosis was also a challenge. Ultimately, the longitudinal and careful evaluation and tracking of symptoms led to the conclusion that the perceptual disturbances were not due to bipolar disorder with psychotic features. WA’s presentation was consistent with DSM‐5 criteria for HPPD, as his persistent visual phenomena resembled experiences associated with prior hallucinogen exposure and caused distress. This was an important distinction given the decision of whether or not to proceed with antipsychotic medication or not. WA’s prior cannabis use may have increased his susceptibility to persistent perceptual disturbances, which is consistent with reports linking cannabis exposure to exacerbation of HPPD symptoms [9].

Furthermore, there is little known about the dose, frequency, and duration of LSD exposure that is necessary for the development of HPPD. HPPD was not considered early on because of the assumption that the exposure was not enough to precipitate the disorder. WA had two LSD exposures during the early onset of mania, which brings up the question of the vulnerability of his brain during that time. Research on how substances impact the brain during vulnerable and critical periods is still a poorly understood topic, although there is some emerging literature. The majority of the research is oriented around cannabis use, and there is less concerning LSD and other hallucinogens. One study found that visual symptoms in HPPD are reported to be higher in adolescents (73.5%) than in adults (34.2%) [10].

This case also exemplifies the importance of psychoeducation about risk factors. WA’s mother had a brief psychotic disorder, as noted earlier. Psychosis has a strong familial and heritable component, and it is important that in families and communities with a history of psychosis, patients are educated on what risk factors are so that they can make informed decisions.

The interaction between WA’s comorbid diagnoses and HPPD is also important to consider. These additional diagnoses may have potentially exacerbated HPPD symptoms, and vice versa. Given that he had psychotic perceptual disturbances as well as HPPD‐related perceptual disturbances, the clinical treatment was challenging in that he required different interventions to manage both.

As aforementioned, the neurobiological mechanisms underlying HPPD remain incompletely understood. Proposed mechanisms of HPPD include the dysfunction of GABAergic inhibitory pathways and abnormalities in thalamocortical visual processing circuits, which may contribute to persistent visual symptoms such as visual snow, halos, and afterimages. These models may help explain why WA’s visual disturbances persisted long after cessation of LSD use and remained relatively independent of fluctuations in mood symptoms and psychosis [1, 6].

The coexistence of bipolar disorder may have also influenced the expression and persistence of HPPD symptoms. Although the relationship between mood disorders and HPPD remains poorly understood, bipolar disorder has been associated with alterations in sensory processing, cortical excitability, and neurotransmitter regulation involving serotonergic and GABAergic systems. It is therefore possible that underlying neurobiological vulnerabilities associated with bipolar disorder increased susceptibility to persistent perceptual disturbances following hallucinogen exposure. Alternatively, heightened emotional distress and anxiety associated with mood symptoms could have amplified the subjective awareness and functional impact of visual phenomena [1, 6].

The chronic nature of his symptoms, including visual snow, light trails, and halos, suggests a diagnosis of HPPD type II, which is typically more severe, long‐term, and sometimes refractory to treatment. While WA does not meet all the criteria for one diagnosis or the other, his symptoms lean more toward HPPD type II without detachment and derealization and other nonvisual side effects. Little is understood about the long‐term management of both types of HPPD, and even less is known about the long‐term course of HPPD in the setting of other serious mental illnesses.

Despite the persistence of his daily visual symptoms, WA’s journey towards acceptance and improved coping mechanisms points to a meaningful outcome with the support from his clinical team. This case reveals the importance of longitudinal evaluation in order to understand and observe how symptoms come and go with various interventions and to maintain diligence in evolving case formulation. WA’s ability to progress in school, career, and social domains, despite ongoing visual disturbances, was aided by his continuous therapy and extensive psychoeducation about how to make sense of his experiences as they relate to symptoms and diagnoses. A significant takeaway from this case is that while symptom resolution may not always be fully achievable, helping patients build resilience by giving them an opportunity to speak about their experiences can significantly enhance their quality of life. Clinicians and psychiatrists should be vigilant in recognizing HPPD in patients presenting with atypical visual phenomena, particularly those with other psychiatric vulnerabilities.

Recent trials have demonstrated promising results for LSD‐assisted treatment of generalized anxiety disorder and depression [7, 8]. As psychedelic‐assisted therapies gain increasing attention within psychiatry, clinicians will likely encounter a growing number of patients with prior therapeutic or recreational exposure to LSD. This case emphasizes the importance of recognizing HPPD as a potentially chronic complication, distinguishing it from primary psychotic disorders, and providing longitudinal support when symptoms persist. A greater understanding of the epidemiology, risk factors, and treatment of HPPD will be essential as psychedelic therapies continue to move toward broader clinical implementation.

## Funding

No funding was received for this manuscript.

## Ethics Statement

Institutional Review Board approval was not required for this case report, according to institutional policy. This case report was prepared in accordance with the CARE (CAse REport) guidelines.

## Consent

The authors obtained written informed consent from the patient for the publication of this case report and accompanying clinical information.

## Conflicts of Interest

The authors declare no conflicts of interest.

## Data Availability

The data that support the findings of this study are available upon request from the corresponding author. The data are not publicly available due to privacy or ethical restrictions.
